# Comparative Immunogenicity of the 2014–2015 Northern Hemisphere Trivalent IIV and LAIV against Influenza A Viruses in Children

**DOI:** 10.3390/vaccines7030087

**Published:** 2019-08-12

**Authors:** Jann Catherine Ang, Biao Wang, Joanne J.F. Wang, Peter Yu Fan Zeng, Florian Krammer, Brian J. Ward, Margaret L. Russell, Mark Loeb, Matthew S. Miller

**Affiliations:** 1 Michael G. DeGroote Institute for Infectious Disease Research, McMaster Immunology Research Centre, Department of Biochemistry and Biomedical Sciences, McMaster University, Hamilton, ON L8S 4L8, Canada; 2 Department of Pathology and Molecular Medicine, McMaster University, Hamilton, ON L8S 4L8, Canada; 3 Department of Microbiology, Icahn School of Medicine at Mount Sinai, New York, NY 10029, USA; 4 Research Institute of McGill University Health Centre, Montreal, QC H3H 2R9, Canada; 5 Department of Community Health Sciences, Cumming School of Medicine, University of Calgary, Calgary, AB T2N 1N4, Canada

**Keywords:** influenza virus, inactivated influenza vaccine, live-attenuated influenza vaccine, childhood vaccination, antibodies, hemagglutinination inhibition, mucosal IgA, correlates of protection

## Abstract

Both inactivated influenza vaccines (IIV) and live-attenuated influenza vaccines (LAIV) have been recommended for administration to children. Children are a high-risk group for severe influenza, and a major source of transmission. Therefore, prevention of infection by vaccination is particularly important. However, efficacy and immunogenicity of these vaccines are known to vary by season and geographic location. We compared the immunogenicity of the 2014–2015 Northern Hemisphere trivalent IIV and LAIV against influenza A virus in Canadian Hutterite children aged 2 to 17 using hemagglutination inhibition (HAI) assays, and enzyme-linked immunosorbent assays to measure hemagglutinin-specific serum IgA and mucosal IgA. Both vaccine formulations induced significant increases in HAI titers against H1N1 and H3N2 vaccine strains. Serum IgA titers against H3N2 were significantly boosted by both IIV and LAIV, while only IIV induced a significant increase in serum IgA specific to the H1N1 vaccine strain. While HAI titers correlated with protection conferred by IIV, mucosal IgA titers correlated with protection conferred by LAIV (mucosal IgA titers could not be established as a correlate for IIV due to sample size limitations). IIV and LAIV were previously reported to be equally efficacious in this cohort, although the immunogenicity of IIV was generally superior.

## 1. Introduction

Seasonal influenza virus epidemics continue to cause millions of hospitalizations, and hundreds of thousands of deaths globally each year [1]. Attack rates for influenza virus infections are consistently highest among children, and those under 5 years of age are considered to be especially vulnerable to severe outcomes [2]. Indeed, a recent systematic analysis estimated that influenza virus infections are responsible for a worldwide average of 870,000 hospitalizations of children <5 years old each year [3]. Furthermore, children are also thought to be a significant source of viral transmission [4,5,6,7]. Seasonal vaccination continues to be the most effective way to prevent influenza virus infection, and many countries have adopted government-sponsored influenza immunization programs to protect their populations.

While inactivated influenza vaccines (IIV) formulations have been available for several decades, the first live-attenuated influenza vaccines (LAIV) widely approved for use in humans was manufactured by MedImmune (FluMist^®^/Fluenz™) and became available in 2003 [8]. In several early randomized clinical trials, trivalent LAIV demonstrated superior efficacy relative to trivalent inactivated influenza virus vaccine (TIV) in children [9,10]. As a result, the U.S. Advisory Committee on Immunization Practices (ACIP) preferentially recommended the use of LAIV for healthy children aged 2 to 8 years during the 2014–2015 season [11]. This preferential recommendation was lifted during the 2015–2016 season, when the ACIP advised that either IIV or LAIV was appropriate for healthy individuals 2 through 49 years [12]. However, in their 2016–2017 report, the ACIP recommended against using the use of LAIV, citing data for the U.S. Vaccine Effectiveness Network that indicated no significant vaccine effectiveness of quadrivalent LAIV against all influenza A and B strains for children aged 2 to 17 [13]. In contrast, a recent cluster randomized blinded trial conducted in children aged 3 to 15 years by members of this group reported equivalent efficacy of IIV and LAIV across the three influenza seasons spanning October 2012 to May 2015 for all strains tested [14]. Most recently, the ACIP has again recommended quadrivalent LAIV for use in populations for whom it is appropriate, but without preference relative to IIV [15].

Here, we compare the immunogenicity of IIV and LAIV after vaccination of Hutterite children with the 2014–2015 Northern Hemisphere trivalent vaccines. Hemagglutination inhibition (HAI) titers, serum IgA endpoint titers, and mucosal IgA titers against the influenza A virus (IAV) components (H1N1 and H3N2) of the vaccines were evaluated.

## 2. Materials and Methods

### 2.1. Study Participants

In this study, we assessed 618 healthy Hutterite children and adolescents aged 3 to 15 years in the 2014–2015 flu season who participated in a cluster randomized clinical trial (ClinicalTrials.gov: NCT01653015) that compared the direct and indirect protectiveness of trivalent IIV and trivalent LAIV, as reported previously [14]. The 2014–2015 season was chosen because this is the only season during which both serum and mucosal samples were collected. In this trial, participants were randomly assigned by colonies to two study groups—IIV group and LAIV group. Healthy children and adolescents aged 3 to 15 years in these two study groups received either IIV or LAIV depending on the group to which their Hutterite colony was randomized. In both LAIV and IIV colonies, previously unvaccinated children aged less than 9 years of age at the time of immunization received a second dose of the same vaccine four weeks following the first dose. The administration of each vaccine formulation occurred over a similar time-frame (average vaccination date for IIV was 2014-11-21; average vaccination date for LAIV was 2014-11-22). For the current study, we used previously obtained specimens from 618 children and adolescent participants from this trial. Of these, 278 were from IIV group and 340 from LAIV group. The IAV antigens included in the both IIV and LAIV were A/California/7/2009 (H1N1) pdm09-like virus, A/Texas/50/2012 (H3N2)-like virus, and B/Massachusetts/2/2012-like virus, all of which were well-matched with circulating strains in Canada during the 2014–2015 influenza season.

### 2.2. Sample Collection

Blood samples were drawn from participants at baseline and between 3 and 5 weeks post-vaccination. For previously unvaccinated children under 9 years of age who received two doses of vaccine, samples were collected after the second dose. Active surveillance was conducted twice weekly during the influenza season (28 December 2014, to 20 May 2015) and those with two or more signs or symptoms compatible with influenza were tested by RT-PCR of nasal swabs. We collected nasal swabs to test for mucosal IgA antibodies at baseline and three weeks after vaccination with either IIV or LAIV.

### 2.3. HAI

The HAI assay was performed as previously described using turkey erythrocytes and reference antigens for A/California/07/2009 (H1N1) pdm09-like virus (Cal/09 H1N1) and A/Texas/50/2012 (H3N2)-like (Tex/50 H3N2) [16].

### 2.4. Enzyme-Linked Immunosorbent Assays (ELISAs)

ELISAs were performed on 96-well Nunc-immuno maxisorp plates (Thermo Scientific, Waltham, MA, USA). The plates were coated overnight with 2 ug/mL per well of purified recombinant HA (A/California/07/2009 H1 or A/Texas/50/2012 H3) in bicarbonate–carbonate coating buffer (100 mM, pH 9.4). The plates were blocked with 5% skim milk for 1 h. Serum samples were serially diluted starting with 1:50 dilution in 5% skim milk and then incubated for 1 h at room temperature. Mucosal samples were undiluted and were incubated for 1 h at room temperature. Plates were washed three times with PBS-T (0.1% Tween 20). Secondary goat anti-human IgA-horseradish peroxidase (HRP) (Santa Cruz Biotechnology, Dallas, TX, USA) at 1:4000 dilution was added in 5% skim milk for 1 h at room temperature, followed by 3x PBS-T wash prior to addition of HRP substrate (Sigmafast OPD, Sigma Aldrich, St. Louis, MO, USA). Reactions were stopped after 10 min by the addition of 3 M HCl, and the optical densities were read at 490 nm on a Spectramax I3 (Molecular Devices, San Jose, CA, USA). Endpoint titers of serum IgA were defined as the lowest dilution whose optical density remained three standard deviations above the mean of negative control wells. Since mucosal IgA could only be reliably measured using undiluted samples, only normalized optical density (O.D.) values were reported. We chose to favor specificity over sensitivity, and therefore, normalization was performed by subtracting the mean O.D. plus three standard deviations of blank samples from each experimental sample.

### 2.5. Statistical Analyses

We compared pre- and post-vaccination antibody titers in terms of three types of antibody measures through paired t tests for both the IIV and LAIV groups. A Mann–Whitney U test was used to compare the changes of the three antibody titers between the IIV and LAIV groups. We used linear regression to estimate the associations between antibody changes and their related factors. A backward stepwise selection method was used to determine the variables in the final model. Finally, we evaluated the correlation of protection against influenza by calculating protective effectiveness using Cox’s proportional hazards model. The protective effectiveness was defined as (1-hazard ratio) X 100%. All analyses were performed in R version 3.4.2.

## 3. Results

### 3.1. Participant Characteristics

The characteristics of participants in the IIV group and LAIV were similar (Table 1). The mean age and standard deviation (SD) of participants was 9.3 (3.3) years in the IIV group and 9.4 (3.2) in the LAIV group. Further, 54.3% of the participants in IIV group were female, compared to 52.1% in LAIV group. We detected 11 (3.9%) PCR-confirmed H3N2 cases in the IIV group and 8 (2.4%) in the LAIV group. H1N1 cases were not detected this season in either group, consistent with the known dominance of H3N2 [17].

### 3.2. Serum HAI Responses to IIV and LAIV

**Figure 1:**
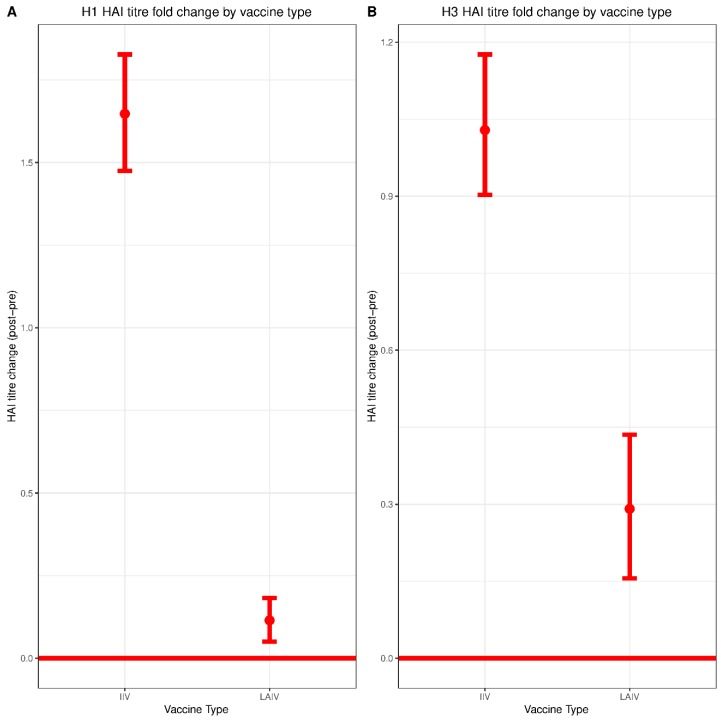
H1N1 and H3N2 pre/post-vaccination hemagglutination inhibition (HAI) titer change by vaccine type. HAI assays were performed against (**A**) Cal/09 H1N1 and (**B**) Tex/50 H3N2 viruses. Log_2_ transformed changes in HAI titer from pre-vaccination to post-vaccination were plotted for each vaccine formulation. Live-attenuated influenza vaccines (LAIV), n = 340; inactivated influenza vaccines (IIV), n = 278. Significance of HAI titer change within each vaccine group was evaluated by paired Student *t* test. Differences between vaccine groups (IIV vs. LAIV) were assessed using Mann–Whitney U Test.

An HAI titer of 40 or greater is a longstanding surrogate correlate of protection against influenza virus infection, although the limitations of this measure have been widely acknowledged, along with the need for alternative correlates [18]. We therefore began by comparing pre- and post-vaccination HAI titers between the IIV group and the LAIV group. In the IIV group, the mean (SD) log transformed HAI titer value against Cal/09 H1N1 was 4.67 (2.07) pre-vaccination and 6.32 (1.59) for post-vaccination, corresponding to a statistically significant increase in serum HAI titers of 1.64 (95% CI: 1.48–1.82, *p* < 0.01) (Figure 1A). In comparison, the mean (SD) log transformed HAI titer against Tex/50 H3N2 was 5.29 (1.69) pre-vaccination and 6.32 (1.14) post-vaccination—a statistically significant increase of 1.03 (95% CI: 0.89–1.17, *p* < 0.01) (Figure 1B). The underlying distributions of changes in titer for each group are shown in Supplementary Figure S1.

In the LAIV group, the mean (SD) log transformed HAI titer against Cal/09 H1N1 was 4.17 (1.91) pre-vaccination and 4.29 (1.87) post-vaccination. While this increase of 0.11 was statistically significant (95% CI: 0.05–0.18, *p* < 0.01), it was substantially lower than that observed for the children vaccinated with IIV (1.64) (Figure 1A). In LAIV group, the mean (SD) log transformed HAI titer against Tex/50 H3N2 was 4.23 (1.83) pre-vaccination and 4.52 (1.76) post-vaccination. There was a statistically significant increase of 0.29 (95% CI: 0.15–0.43, *p* < 0.01), which again was lower than that observed for participants who received IIV (Figure 1B). Overall, the post-vaccination changes in HAI titers against Cal/09 H1N1 and Tex/50 H3N2 in the IIV group were significantly higher than those observed for the LAIV group (*p* < 0.01). Furthermore, the elevated pre-vaccination titers observed in the IIV group (Table 1) likely reflect the fact that most of the participants in that group had also received IIV in the previous two seasons. Pre-vaccination HAI titers were a relatively strong negative correlate of HAI titer change (ΔHAI) in the IIV group. This negative correlation was also detected in the LAIV group, though it was substantially weaker (Supplementary Figure S4A,B).

### 3.3. Serum IgA Responses to IIV and LAIV

**Figure 2:**
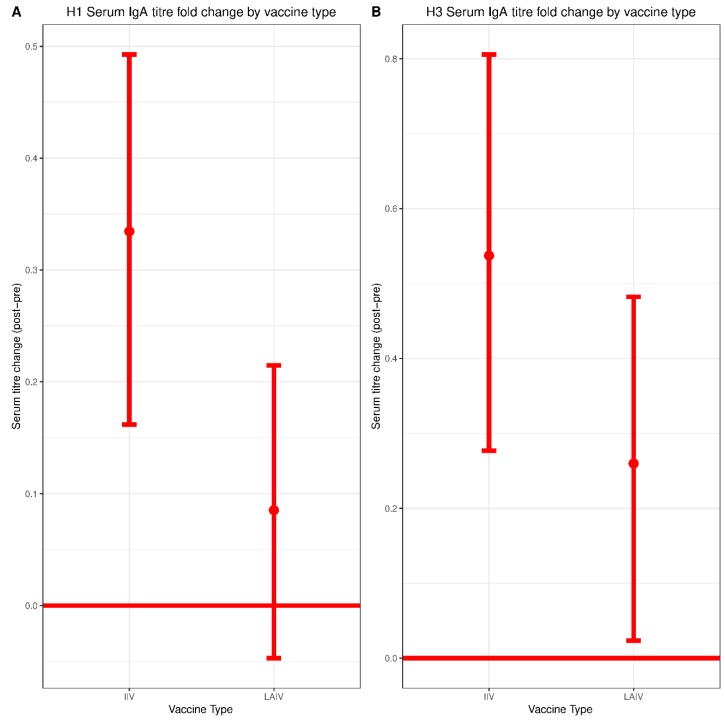
H1 and H3 pre/post-vaccination serum IgA endpoint titer change by vaccine type. Endpoint enzyme-linked immunosorbent assays (ELISA) assays were performed to measure titers of serum-derived IgA using (**A**) recombinant Cal/09 H1 protein or (**B**) recombinant Tex/50 H3 protein. Log_2_ transformed changes in endpoint IgA titer from pre-vaccination to post-vaccination were plotted for each vaccine formulation. LAIV, n = 340; IIV, n = 278. Significance of IgA endpoint titer change within each vaccine group was evaluated by paired Student *t* test. Differences between vaccine groups (IIV vs. LAIV) were assessed using Mann–Whitney U Test.

Local IgA responses have generated substantial interest for their potential role in LAIV-mediated protection [19,20,21]; however, serum IgA responses have been relatively understudied. Therefore, we specifically interrogated serum IgA titers before and after vaccination with IIV and LAIV. The mean (SD) log transformed serum IgA endpoint titer against Cal/09 H1N1 in the IIV group was 2.90 (2.51) pre-vaccination and 3.23 (2.74) post-vaccination, corresponding to a statistically significant increase of 0.33 (95% CI: 0.17–0.50, *p* < 0.01) (Figure 2A). The mean (SD) log transformed serum IgA endpoint titer against Tex/50 H3N2 in the IIV group was 4.25 (2.76) pre-vaccination and 4.79 (2.79) post-vaccination, representing a statistically significant increase of 0.54 (95% CI: 0.27–0.81, *p* < 0.01) (Figure 2B). The underlying distributions of changes in titer for each group are shown in Supplementary Figure S2.

The mean (SD) log transformed serum IgA endpoint titer against Cal/09 H1N1 in the LAIV group was 2.88 (2.27) pre-vaccination and 2.96 (2.36) post-vaccination. This increase was not statistically significant (0.09, 95% CI: −0.05–0.22, *p* = 0.22) (Figure 2A). The mean (SD) log transformed serum IgA titer value against Tex/50 H3N2 in the LAIV group was 4.12 (2.50) pre-vaccination and 4.38 (2.59) post-vaccination. There was a statistically significant increase of 0.26 (95% CI: 0.02–0.50, *p* = 0.03) (Figure 2B). While the boost in serum IgA was not significantly different between the IIV and LAIV groups, there was a trend toward greater boosts in those who received IIV.

### 3.4. Mucosal Responses to IIV and LAIV

**Figure 3:**
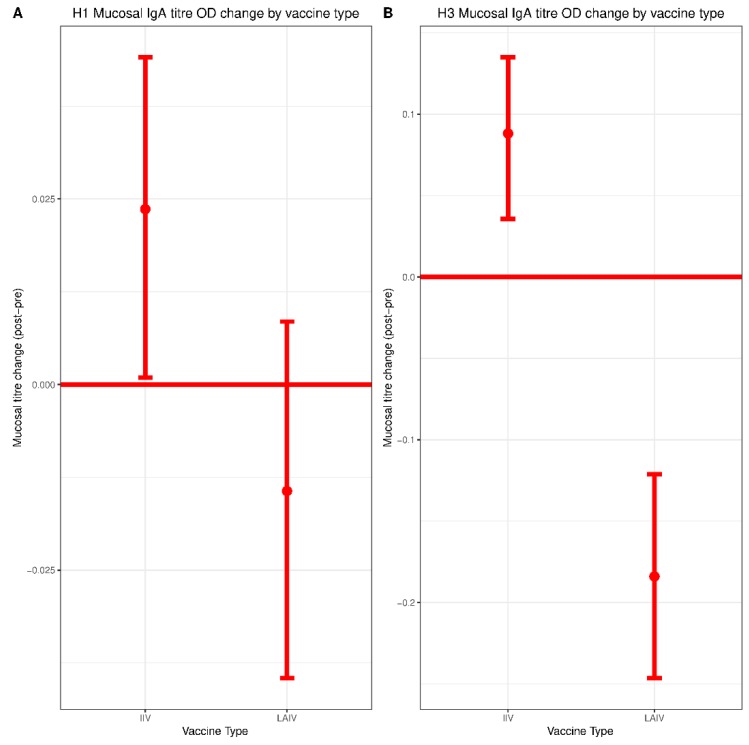
H1 and H3 pre/post-vaccination mucosal IgA titer change by vaccine type. ELISA assays were performed to measure titers of mucosal IgA collected by nasal swab using (**A**) recombinant Cal/09 H1 protein or (**B**) recombinant Tex/50 H3 protein. Normalized optical density from pre-vaccination to post-vaccination were plotted for each vaccine formulation. LAIV, n = 340; IIV, n = 278. Significance of mucosal IgA titer change within each vaccine group was evaluated by paired Student *t* test. Differences between vaccine groups (IIV vs. LAIV) were assessed using Mann–Whitney U Test.

The mean (SD) mucosal IgA titer against Cal/09 H1N1 from the IIV group was 0.08 (0.16) pre-vaccination and 0.10 (0.14) post-vaccination, representing a statistically significant increase of 0.02 (95% CI: 0.00–0.05, *p* = 0.03) (Figure 3A). The mean (SD) mucosal IgA titer value against Tex/50 H3N2 in the IIV group was 0.21 (0.33) pre-vaccination and 0.29 (0.30) post-vaccination, a statistically significant increase of 0.09 (95% CI: 0.04–0.14, *p* < 0.01) (Figure 3B).

The mean (SD) mucosal IgA endpoint titer against Cal/09 H1N1 in the LAIV group was 0.11 (0.25) pre-vaccination and 0.10 (0.14) post-vaccination, corresponding to a non-significant decrease of −0.01 (95% CI: −0.04–0.05, *p* = 0.23) (Figure 3A). The mean (SD) mucosal IgA titer against Tex/50 H3N2 in the LAIV group was 0.38 (0.50) pre-vaccination and 0.21 (0.25) post-vaccination, a statistically significant decrease of −0.18 (95% CI: −0.25–−0.11, *p* < 0.01) (Figure 3B). Individual data points depicting the distribution of changes in titer for each subtype are shown in Supplementary Figure S3.

Pre-vaccination mucosal IgA titers against Tex/50 H3N2 were substantially higher in the LAIV group (Table 1), which was likely a result of most participants in this group having received LAIV in the previous two seasons. The change in mucosal IgA titers against Cal/09 H1N1 post-vaccination did not differ significantly between IIV and LAIV groups. However, there was a significant difference in post-vaccination antibody titers against Tex/50 H3N2, with the IIV group demonstrating a superior response. A weak, but significant negative correlation was observed for the relationship between pre-vaccination mucosal IgA titers and mucosal IgA titer change against H3 (Supplementary Figure S4E,F) Since pre-existing antibody titers are thought to inhibit responses against LAIV, we performed a sub-analysis of individuals whose mucosal IgA titers were undetectable in pre-vaccination samples, but became detectable post-vaccination (i.e., mucosal IgA conversion). Both IIV and LAIV were similarly capable of inducing mucosal IgA conversion (Supplementary Figure S5A). The proportion of participants with detectable levels of IgA post-vaccination was also similar between groups (Supplementary Figure S5B).

### 3.5. Variables Associated with Antibody Response to Vaccination

As shown in Table 2, the magnitude of change in HAI titers against Cal/09 H1N1 was significantly associated with vaccine type (i.e., IIV vs. LAIV), pre-vaccination titer, and age. The change of serum IgA titers was significantly associated with vaccine type and pre-vaccination titer level, but not age. Finally, the change in mucosal IgA titer was only significantly associated with pre-vaccination titer level.

The change of HAI titers against Tex/50 H3N2 was significantly associated with vaccine type (i.e., LAIV vs. IIV), pre-vaccination titer, and age. The change of serum IgA titers was significantly associated with vaccine type and pre-vaccination titer. The change of mucosal IgA titer was significantly associated with vaccine type and pre-vaccination titer (Table 3).

### 3.6. Correlates of Protection against H3N2 Infection

We only detected H3N2 cases in the study participants, consistent with the known dominance of H3N2 circulation during the 2014–2015 season. Therefore, the correlates of protection analysis were only performed for the H3N2 virus.

For HAI titers, protective effectiveness estimates in the IIV group were statistically significant at titer values of 1:80 and 1:160. The estimates at these two titer values were similar (77% vs. 76%) (Table 4). HAI titer was not a correlate of protection in the LAIV group.

Serum IgA titers did not correlate with protection in either the IIV group or the LAIV group (Table 5). For mucosal IgA titers, protective effectiveness estimates for the LAIV group were statistically significant at all cut-off values. The estimates at 0.0001, 0.001, and 0.01 were similar (88% vs. 87% vs. 88%) (Table 6). We were unable to produce estimates for IIV group due to the smaller sample size.

## 4. Discussion

Children represent an extremely important group when it comes to the prevention of influenza virus infection by vaccination due to their elevated risk for severe illness, and their role as a major source of viral transmission [22,23]. Indeed, vaccination of children alone can have profound effects on the ‘herd/population immunity’ elicited by influenza vaccines [22]. Therefore, ensuring that children receive influenza vaccine formulations that provide optimal vaccine efficacy is of paramount importance.

Despite observational data from the U.S. Vaccine Effectiveness network that led ACIP to recommend against the use of LAIV during the 2016–2017 influenza season, members of our group previously published the results of a cluster-randomized control trial in which the efficacy of IIV and LAIV was equivalent across the three influenza seasons spanning October 2012 to May 2015 for all strains present in the trivalent vaccine [14]. However, the immunogenicity of IIV and LAIV in this cohort had not been analyzed, nor were immunological correlates of protection identified. Therefore, we performed an immunogenicity analysis of the humoral responses of that same cohort using serum and mucosal samples collected from the 2014–2015 influenza season (since this was the only season during which both serum and mucosal samples were collected).

Both IIV and LAIV induced significant increases in serum HAI titers against both H1N1 and H3N2 however, the magnitude of seroconversion induced by IIV was much greater—consistent with prior studies [24]. HAI titers of 1:80 and 1:160 correlated with protection against H3N2 infection for the group immunized with IIV. This observation is in line with previous studies reporting HAI titers of greater than 1:40 as being required for protection in children immunized with IIV [16,25].

IIV recipients experienced a small but significant boost in mucosal IgA titers against both H1N1 and H3N2 vaccine components. No increase in mucosal IgA was observed in the LAIV group. However, consistent with an earlier H1N1 challenge study of children vaccinated with LAIV, detection of any mucosal IgA correlated with protection against H3N2 infection [20]. Given the similarity in mucosal IgA titers when comparing IIV and LAIV groups, as well as the fact that the IIV group tended to mount stronger post-vaccination responses, it is possible that mucosal IgA might also serve as a correlate of protection for IIV. However, limitations in sample size precluded the generation of estimates for IIV in our model. This issue should be explored more thoroughly in future studies of larger cohorts.

The lack of a detectable boost in mucosal IgA titers in the LAIV group was surprising, since LAIV has been reported to boost mucosal IgA titers in several previous studies [21,26,27,28]. Several possibilities might explain this discrepancy. Firstly, pre-vaccination mucosal IgA titers against H3N2 were much higher in the LAIV group when compared to the IIV group. This likely reflects the fact that most children in the LAIV group had also been vaccinated with LAIV for the previous two seasons. High pre-vaccination antibody titers are known to correlate negatively with response to vaccination, which we also observed in response to H3—although the effect was modest [29]. This could also be due to differences in sampling methodologies. Most prior studies measured mucosal IgA collected in nasal washes whereas we detected mucosal IgA collected in nasal swabs, which are likely to have lower concentrations of antibody. Indeed, we were only able to reliably measure mucosal IgA above our limit of detection using undiluted samples, which precluded titration-based measurements. Notably, serum IgA did not correlate with protection in either the IIV or the LAIV group, highlighting the distinct composition of serum and mucosal IgA pools.

The influence of pre-existing immunity on the vaccine-induced responses during the 2014–2015 influenza season is also an important consideration in this study—since most of the participants had been enrolled in the study and vaccinated for each of the two previous seasons [14]. Indeed, considerable pre-vaccination HAI titers against H1N1 and H3N2 vaccine strains were observed in both the IIV and LAIV groups. Pre-existing immunity has, in certain instances, been shown to limit responses to LAIV—probably by restricting replication of the attenuated virus [28,30]. High titers of pre-existing antibodies have also been identified as a negative correlate of serological responses in multiple systems biology studies of influenza virus vaccination [29,31]. Consistent with these reports, we found that pre-existing antibody titers were negatively correlated with HAI responses, and that this relationship was strongest in the IIV group. A weak, but significant negative correlation between H3 pre-vaccination mucosal IgA titers and post-vaccination response was also found for both IIV and LAIV. Interestingly, a study using a mouse model of influenza previously reported that influenza virus-specific B cells are sensitized to viral infection and subsequently, cell death. This led the authors to speculate that killing of these cells during early infection may allow the virus to establish initial replication and delay the memory antibody response [32]. This has never been established in humans, to our knowledge. However, the small (but significant) decreases in mucosal IgA observed only in the LAIV group would be consistent with such a phenomenon and warrants further investigation. Given the modest magnitude of this decrease, it is also possible that while statistically significant (due to the relatively large number of samples analyzed), the decrease is not biologically meaningful.

A notable limitation of this study is that peripheral blood mononuclear cells were not collected and thus, T cell responses were not measured. LAIV is known to effectively boost T cell responses, including cross-reactive T cells that may help to protect against drifted or novel strains [28,33,34]. Granzyme B levels have also been associated with protection against laboratory diagnosed influenza after vaccination of older adults [35]. In addition, microneutralization titers capture a more complete picture of neutralizing antibody responses than HAI assays, and may also be an important correlate of protection. MNTs were not performed here due to limitations in sample availability. This study was performed as a cluster randomized trial. Therefore, cluster effects should, thus, also be considered when interpreting this data. For example, shedding of LAIV between vaccinated and not-yet-vaccinated individuals within the LAIV cluster could influence the kinetics and magnitude of antibody boosting. However, if this were to occur, it did not result in more robust community protection afforded by LAIV in this trial [14]. Furthermore, most children would have received the same vaccine formulation in the previous two influenza seasons.

Taken together, the results of this study provide new insights into the nature of the humoral immune response elicited by IIV and LAIV in children. While vaccine efficacy against all strains of influenza virus were equivalent in this cohort, the immune responses elicited by each vaccine were distinct, as were their respective correlates of protection. By most measures, IIV demonstrated superior immunogenicity compared to LAIV. The data presented here reinforce the notion that the HAI titer suggestive of antibody-mediated protection may be greater than 1:40 in children—and that distinct correlates of protection are required for IIV and LAIV.

## Figures and Tables

**Table 1:** Basic characteristics of the study participants and distributions of pre-and post-vaccination antibody titers.

Variable		Vaccination Type	
		IIV	LAIV
Number of Participants		278	340
Previously Study-Vaccinated		241 (87%)	294 (86%)
Number of Infections (H3N2)		11 (3.9%)	8 (2.4%)
Age, mean(SD)		9.3 (3.3)	9.4 (3.2)
	3–9 years, No. (%)	143 (51.4%)	170 (50%)
	10–15 years, No. (%)	135 (48.6%)	170 (50%)
**Female Sex**		151 (54.3%)	177 (52.1%)
**Pre and Post vaccination antibody titer for H1N1**			
**HAI (log2 titer)**			
	Pre, mean (SD)	4.67 (2.07)	4.17 (1.91)
	Post, mean (SD)	6.32 (1.59)	4.29 (1.87)
**Serum IgA (log2 titer)**			
	Pre, mean (SD)	2.90 (2.51)	2.88 (2.27)
	Post, mean (SD)	3.23 (2.74)	2.96 (2.36)
**Mucosal IgA (OD)**			
	Pre, mean(SD)	0.08 (0.16)	0.11 (0.25)
	Post, mean(SD)	0.10 (0.14)	0.10 (0.14)
**Pre and Post vaccination antibody titer for H3N2**			
**HAI (log2 titer)**			
	Pre, mean (SD)	5.29 (1.69)	4.23 (1.83)
	Post, mean (SD)	6.32 (1.14)	4.52 (1.76)
**Serum IgA (log2 titer)**			
	Pre, mean (SD)	4.25 (2.76)	4.12 (2.50)
	Post, mean (SD)	4.79 (2.79)	4.38 (2.59)
**Mucosal IgA (OD)**			
	Pre, mean (SD)	0.21 (0.33)	0.38 (0.50)
	Post, mean (SD)	0.29 (0.30)	0.21 (0.25)

**Table 2:** Variables associated with antibody response to A/California/07/2009-like H1N1.

Antibody Measures							
Variable		HAI Titer Change (log)		Serum IgA Titer Change (log)		Mucosal IgA Change (OD, ^0.3)	
		*β* (95% CI)	*p*-Value	*β* (95% CI)	*p*-Value	*β* (95% CI)	*p*-Value
**Vaccine Type**	LAIV vs. IIV	−1.66 (−1.81 to −1.50)	<0.01	−0.25 (−0.46 to −0.04)	0.02		
**Pre vaccination titer**	Per 1-unit	−0.25 (−0.29 to −0.21)	<0.01	−0.09 (−0.14 to −0.05)	<0.01	−0.71 (−0.78 to −0.64)	<0.01
**Age**	Per 1 year	−0.03 (−0.06 to −0.01)	<0.01				

Mucosal IgA OD was transformed by X^0.3 (Tukey transformation).

**Table 3:** Variables associated with antibody response to A/Texas/50/2012-like H3N2.

Antibody Measures							
Variable		HAI Titer Change (Log)		Serum IgA Titer Change (Log)		Mucosal IgA Change (OD, ^0.3)	
		*β* (95% CI)	*p*-Value	*β* (95% CI)	*p*-Value	*β* (95% CI)	*p*-Value
**Vaccine Type**	LAIV vs. IIV	−0.29 (−0.34 to −0.24)	<0.01	−0.33 (−0.66 to 0.00)	0.05	−0.10 (−0.14 to −0.06)	<0.01
**Pre vaccination titer**	Per 1-unit	−0.30 (−0.34 to −0.25)	<0.01	−0.35 (−0.41 to −0.28)	<0.01	−0.94 (−0.10 to −0.87)	<0.01
**Age**	Per 1 year	−0.05 (−0.07 to −0.02)	<0.01				

Mucosal IgA OD was transformed by X^0.3 (Tukey transformation).

**Table 4:** HAI protectiveness against H3N2.

Cut-Off	Titer Value	IIV			LAIV		
		Protectiveness (95% CI)	*p* Value	Infected above the Cut-Off ^†^	Protectiveness (95% CI)	*p* Value	Infected above the Cut-Off ^‡^
3	20			11	60 (−180 to 94)	0.36	6
4	40	81 (−41 to 98)	0.10	10	49 (−392 to 95)	0.56	5
5	80	77 (44 to 90)	<0.01	9	21 (−373 to 87)	0.80	4
6	160	76 (7 to 94)	0.04	5	−52 (−654 to 69)	0.61	3
7	320	39 (−34 to 72)	0.22	4	−187 (−1529 to 49)	0.23	2
8	640	40 (−164 to 87)	0.50	1	−443 (−3794 to 24)	0.09	1

^†^ Total infected = 11; ^‡^ Total infected = 8.

**Table 5:** Protectiveness of serum IgA against H3N2.

Cut-Off	Titer Value	IIV			LAIV		
		Protectiveness (95% CI)	*p* Value	Infected above the Cut-Off ^†^	Protectiveness (95% CI)	*p* Value	Infected above the Cut-Off ^‡^
2	50	11 (−245 to 77)	0.86	10	13 (−482 to 87)	0.89	7
3	100	25 (−91 to 70)	0.55	7	−41 (−562 to 70)	0.66	6
4	200	41 (−100 to 83)	0.40	5	15 (−218 to 77)	0.82	4
5	400	63 (−53 to 91)	0.17	3	25 (−166 to 79)	0.66	3
6	800	40 (−140 to 85)	0.47	3	−24 (−342 to 65)	0.74	3
7	1600	41 (−111 to 83)	0.42	2	−22 (−292 to 62)	0.74	2
8	3200	11 (−226 to 76)	0.86	2	−115 (−567 to 31)	0.18	2
9	6400	−20 (−366 to 69)	0.80	2	−9 (−377 to 75)	0.91	1
10	12,800	−202 (−1183 to 29)	0.14	2			

^†^ Total infected = 11; ^‡^ Total infected = 8.

**Table 6:** Protectiveness of mucosal IgA against H3N2.

Cut-Off -Titer Value	IIV			LAIV		
	Protectiveness (95% CI)	*p* Value	Infected above the Cut-Off ^†^	Protectiveness (95% CI)	*p* Value	Infected above the Cut-Off ^‡^
0.0001	N.D.	N.D.	10	88 (54 to 97)	0.00	4
0.001	N.D.	N.D.	10	87 (48 to 97)	0.00	4
0.01	N.D.	N.D.	10	88 (18 to 98)	0.03	3
0.1	N.D.	N.D.	10	100 (100 to 100)	0.00	0
1	100 (100 to 100)	0.00	0	100 (100 to 100)	0.00	0

N.D. = not determined. In these cases, protectiveness estimates could not be generated by the model due to low sample size. ^†^ Total infected = 11; ^‡^ Total infected = 8.

## Supplementary Materials

The following are available online at https://www.mdpi.com/2076-393X/7/3/87/s1, Figure S1: H1N1 and H3N2 pre/post-vaccination HAI titer change distribution by vaccine type, Figure S2: H1 and H3 pre/post-vaccination serum IgA endpoint titer change distribution by vaccine type, Figure S3: H1 and H3 pre/post-vaccination mucosal IgA titer change distribution by vaccine type, Figure S4: Relationship between pre-vaccination antibody titers and post-vaccination response, Figure S5: Binary quantification of mucosal IgA detection.

## References

[1] WHO|Influenza. http://www.who.int/mediacentre/factsheets/2003/fs211/en/.

[2] Thompson W.W., Shay D.K., Weintraub E., Brammer L., Cox N., Anderson L.J., Fukuda K. (2003). Mortality associated with influenza and respiratory syncytial virus in the United States. JAMA.

[3] Lafond K.E., Nair H., Rasooly M.H., Valente F., Booy R., Rahman M., Kitsutani P., Yu H., Guzman G., Coulibaly D. (2016). Global Role and Burden of Influenza in Pediatric Respiratory Hospitalizations, 1982–2012: A Systematic Analysis. PLoS Med..

[4] Foy H.M., Cooney M.K., Allan I. (1976). Longitudinal studies of types A and B influenza among Seattle schoolchildren and families, 1968–1974. J. Infect. Dis..

[5] Fox J.P., Hall C.E., Cooney M.K., Foy H.M. (1982). Influenzavirus infections in Seattle families, 1975–1979. I. Study design, methods and the occurrence of infections by time and age. Am. J. Epidemiol..

[6] Monto A.S., Koopman J.S., Longini I.M. (1985). Tecumseh study of illness. XIII. Influenza infection and disease, 1976–1981. Am. J. Epidemiol..

[7] Neuzil K.M., Hohlbein C., Zhu Y. (2002). Illness among schoolchildren during influenza season: Effect on school absenteeism, parental absenteeism from work, and secondary illness in families. Arch. Pediatr. Adolesc. Med..

[8] Administration, U.S.F. and D. CBER Approval Letter, Influenza Virus Vaccine, Live, Intranasal (Flumist). https://web.archive.org/web/20070929154324/http://www.fda.gov/cber/approvltr/inflmed061703L.htm.

[9] Rhorer J., Ambrose C.S., Dickinson S., Hamilton H., Oleka N.A., Malinoski F.J., Wittes J. (2009). Efficacy of live attenuated influenza vaccine in children: A meta-analysis of nine randomized clinical trials. Vaccine.

[10] Belshe R.B., Edwards K.M., Vesikari T., Black S.V., Walker R.E., Hultquist M., Kemble G., Connor E.M. (2007). Live Attenuated versus Inactivated Influenza Vaccine in Infants and Young Children. N. Engl. J. Med..

[11] Grohskopf L.A., Olsen S.J., Sokolow L.Z., Bresee J.S., Cox N.J., Broder K.R., Karron R.A., Walter E.B. (2014). Prevention and Control of Influenza with Vaccines: Recommendations of the Advisory Committee on Immunization Practices (ACIP)—United States, 2014–2015 Influenza Season. Morb. Mortal. Wkly. Rep..

[12] Grohskopf L.A., Sokolow L.Z., Olsen S.J., Bresee J.S., Broder K.R., Karron R.A. (2015). Prevention and Control of Influenza with Vaccines: Recommendation of the Advisory Committee on Immunization Practices, United States, 2015–2016 Influenza Season. Morb. Mortal. Wkly. Rep..

[13] Grohskopf L.A., Sokolow L.Z., Broder K.R., Olsen S.J., Karron R.A., Jernigan D.B., Bresee J.S. (2016). Prevention and Control of Seasonal Influenza with Vaccines. Recommendations of the Advisory Committee on Immunization Practices - United States, 2016–2017 Influenza Season. Morb. Mortal. Wkly. Rep..

[14] Loeb M., Russell M.L., Manning V., Fonseca K., Earn D.J.D., Horsman G., Chokani K., Vooght M., Babiuk L., Schwartz L. (2016). Live Attenuated Versus Inactivated Influenza Vaccine in Hutterite Children. Ann. Intern. Med..

[15] Grohskopf L.A., Sokolow L.Z., Fry A.M., Walter E.B., Jernigan D.B. (2018). Update: ACIP Recommendations for the Use of Quadrivalent Live Attenuated Influenza Vaccine (LAIV4)—United States, 2018–2019 Influenza Season. MMWR. Morb. Mortal. Wkly. Rep..

[16] Wang B., Russell M.L., Brewer A., Newton J., Singh P., Ward B.J., Loeb M. (2017). Single radial haemolysis compared to haemagglutinin inhibition and microneutralization as a correlate of protection against influenza A H3N2 in children and adolescents. Influenza Other Respi. Viruses.

[17] FluWatch Report: December 21, 2014 to January 3, 2015 (Weeks 52–53)—Canada.ca. https://www.canada.ca/en/public-health/services/publications/diseases-conditions/fluwatch-report-december-21-2014-january-3-2015-weeks-52-53.html.

[18] Cox R.J. (2013). Correlates of protection to influenza virus, where do we go from here?. Hum. Vaccin. Immunother..

[19] Hoft D.F., Lottenbach K.R., Blazevic A., Turan A., Blevins T.P., Pacatte T.P., Yu Y., Mitchell M.C., Hoft S.G., Belshe R.B. (2017). Comparisons of the Humoral and Cellular Immune Responses Induced by Live Attenuated Influenza Vaccine and Inactivated Influenza Vaccine in Adults. Clin. Vaccine Immunol..

[20] Belshe R.B., Gruber W.C., Mendelman P.M., Mehta H.B., Mahmood K., Reisinger K., Treanor J., Zangwill K., Hayden F.G., Bernstein D.I. (2000). Correlates of Immune Protection Induced by Live, Attenuated, Cold-Adapted, Trivalent, Intranasal Influenza Virus Vaccine. J. Infect. Dis..

[21] Barría M.I., Garrido J.L., Stein C., Scher E., Ge Y., Engel S.M., Kraus T.A., Banach D., Moran T.M. (2013). Localized mucosal response to intranasal live attenuated influenza vaccine in adults. J. Infect. Dis..

[22] Loeb M., Russell M.L., Moss L., Fonseca K., Fox J., Earn D.J.D., Aoki F., Horsman G., Van Caeseele P., Chokani K. (2010). Effect of Influenza Vaccination of Children on Infection Rates in Hutterite Communities. JAMA.

[23] Iuliano A.D., Roguski K.M., Chang H.H., Muscatello D.J., Palekar R., Tempia S., Cohen C., Gran J.M., Schanzer D., Cowling B.J. (2018). Estimates of global seasonal influenza-associated respiratory mortality: A modelling study. Lancet.

[24] Edwards K.M., Dupont W.D., Westrich M.K., Plummer W.D., Palmer P.S., Wright P.F. (1994). A Randomized Controlled Trial of Cold-Adapted and Inactivated Vaccines for the Prevention of Influenza A Disease. J. Infect. Dis..

[25] Black S., Nicolay U., Vesikari T., Knuf M., Del Giudice G., Della Cioppa G., Tsai T., Clemens R., Rappuoli R. (2011). Hemagglutination Inhibition Antibody Titers as a Correlate of Protection for Inactivated Influenza Vaccines in Children. Pediatr. Infect. Dis. J..

[26] Rose M.A., Zielen S., Baumann U. (2012). Mucosal immunity and nasal influenza vaccination. Expert Rev. Vaccines.

[27] Gorse G.J., Otto E.E., Powers D.C., Chambers G.W., Eickhoff C.S., Newman F.K. (1996). Induction of Mucosal Antibodies by Live Attenuated and Inactivated Influenza Virus Vaccines in the Chronically III Elderly. J. Infect. Dis..

[28] Mohn K.G.-I., Smith I., Sjursen H., Cox R.J. (2018). Immune responses after live attenuated influenza vaccination. Hum. Vaccin. Immunother..

[29] Miller M.S., Palese P. (2014). Peering into the crystal ball: Influenza pandemics and vaccine efficacy. Cell.

[30] Brickley E.B., Wright P.F., Khalenkov A., Neuzil K.M., Ortiz J.R., Rudenko L., Levine M.Z., Katz J.M., Brooks W.A. (2018). The effect of pre-existing immunity on virus detection and immune responses in a phase II randomized trial of a Russian-backbone live attenuated influenza vaccine in Bangladeshi children. Clin. Infect. Dis..

[31] Stacey H., Barjesteh N., Mapletoft J., Miller M. (2018). “Gnothi Seauton”: Leveraging the Host Response to Improve Influenza Virus Vaccine Efficacy. Vaccines.

[32] Dougan S.K., Ashour J., Karssemeijer R.A., Popp M.W., Avalos A.M., Barisa M., Altenburg A.F., Ingram J.R., Cragnolini J.J., Guo C. (2013). Antigen-specific B-cell receptor sensitizes B cells to infection by influenza virus. Nature.

[33] Mohn K.G.I., Zhou F., Brokstad K.A., Sridhar S., Cox R.J. (2017). Boosting of Cross-Reactive and Protection-Associated T Cells in Children After Live Attenuated Influenza Vaccination. J. Infect. Dis..

[34] Eichelberger M.C., Rivers K.H., Ream R., Gao J., Hassantoufighi A.S., Bulte M.R., Straight T.M. (2012). Qualitative Differences in T cell responses to Live, Attenuated and Inactivated Influenza Vaccines. J. Clin. Cell. Immunol..

[35] McElhaney J.E., Ewen C., Zhou X., Kane K.P., Xie D., Hager W.D., Barry M.B., Kleppinger A., Wang Y., Bleackley R.C. (2009). Granzyme B: Correlates with protection and enhanced CTL response to influenza vaccination in older adults. Vaccine.

